# Surgical Treatment with Locoregional Flap for the Nose

**DOI:** 10.1155/2017/9750135

**Published:** 2017-12-24

**Authors:** Marco Marcasciano, Mauro Tarallo, Michele Maruccia, Benedetta Fanelli, Giorgio La Viola, Donato Casella, Lenia Sanchèz Wals, Sergio Ciaschi, Paolo Fioramonti

**Affiliations:** ^1^Department of Surgery “Valdoni”, Unit of Plastic and Reconstructive Surgery, “Sapienza” University of Rome, Rome, Italy; ^2^Department of Emergency and Organ Transplantation, Plastic and Reconstructive Surgery and Burns Unit, University of Bari “Aldo Moro”, Bari, Italy; ^3^Dermatology Unit “Daniele Innocenzi”, Department of Medical and Surgical Sciences and Biotechnologies, Sapienza University of Rome, 04019 Terracina, Italy; ^4^Instituto Nacional de Oncología y Radiobiología, Servicio de Cirugía Reconstructiva, 29 yF Vedado, Plaza de la Revolución, 10400 La Habana, Cuba

## Abstract

Nonmelanotic skin cancers (NMSCs) are the most frequent of all neoplasms and nasal pyramid represents the most common site for the presentation of such cutaneous malignancies, particularly in sun-exposed areas: ala, dorsum, and tip. Multiple options exist to restore functional and aesthetic integrity after skin loss for oncological reasons; nevertheless, the management of nasal defects can be often challenging and the best “reconstruction” is still to be found. In this study, we retrospectively reviewed a total of 310 patients who presented to our Department of Plastic and Reconstructive Surgery for postoncological nasal reconstruction between January 2011 and January 2016. Nasal region was classified into 3 groups according to the anatomical zones affected by the lesion: proximal, middle, and distal third. We included an additional fourth group for complex defects involving more than one subunit. Reconstruction with loco regional flaps was performed in all cases. Radical tumor control and a satisfactory aesthetic and functional result are the primary goals for the reconstructive surgeon. Despite tremendous technical enhancements in nasal reconstruction techniques, optimal results are usually obtained when “like is used to repair like.” Accurate evaluation of the patients clinical condition and local defect should be always considered in order to select the best surgical option.

## 1. Introduction

The nasal pyramid is the most common site for the presentation of head and neck cutaneous malignancies, particularly in sun-exposed areas such as the ala, dorsum, and tip of the nose. Nonmelanoma skin cancers (NMSCs) are mostly slow growing and unlikely to metastasize but represents the most common cancer in the world, with an incidence 18–20 times greater than that of malignant melanoma. Among them, basal cell carcinoma (BCC) is considered the most frequent followed by squamous cell carcinomas (SCCs) [1].

With the increase in the incidence of these neoplasms, dermatologists and plastic surgeons are experiencing a sharp increase in the numbers of patients who need treatment because of the tremendous increase in the incidence of skin cancer [2]. Nose is particularly vulnerable to cutaneous malignancies. A unique anatomy combined with the aesthetic and functional importance makes its reconstruction challenging [3].

Multiple options exist to restore functional and aesthetic integrity after skin loss for oncological reasons (including autogenous, allogenous, and xenogenous tissue transfer, as well as implantation of alloplastic materials); nevertheless, despite tremendous technical enhancements in nasal reconstruction techniques, optimal results are usually obtained when “like is used to repair like” [4].

Since Burget and Menick have further refined nasal reconstruction techniques by introducing aesthetic subunit principles, locoregional flaps continue to play a substantial role in reconstruction of soft tissue and cutaneous defects of the nose [5].

Several recent studies that examine the vascular supply to the head and neck have increased our ability to successfully design locoregional flaps, helping surgeons to prevent ischemia and necrosis [5].

In this study, we retrospectively reviewed a total of 310 patients who presented to our Department of Plastic and Reconstructive Surgery for postoncological nasal reconstruction. We focused on defects that could be reconstructed with locoregional flaps [6].

## 2. Material/Patients and Methods

Respecting the principles of the 1975 Declaration of Helsinki, we collected medical records and photographs of 310 consecutive patients who underwent nasal reconstruction after excision of NMSC, between January 2011 and January 2016.

At clinical examination, anamnesis of the patients and characteristics of the lesion were assessed with regard to age and sex. All the defects were mapped according to the location on the nose and to the subunit principle, defect size, and type of flap involved in nasal reconstruction (Table 1).

Inclusion criteria to the study were as follows: age between 40 and 88 years; a maximum diameter of the lesions ranging between 1.5 and 4 cm; local invasion of superficial nasal layers (skin and subcutaneous tissues); the diagnosis of NMSC was clinically undertaken first and written consent form was signed by all before surgical excision. In all enrolled patients, local treatment was achieved by conventional excision under loupe magnification, removing 3 mm of perilesional clinically safe skin for BCC under 2 cm; 5 mm for BCC between 2 and 4 cm and SCC between 1.5 and 2 cm; 1 cm for SCC between 2 and 4 cm [7].

**Figure 1:**
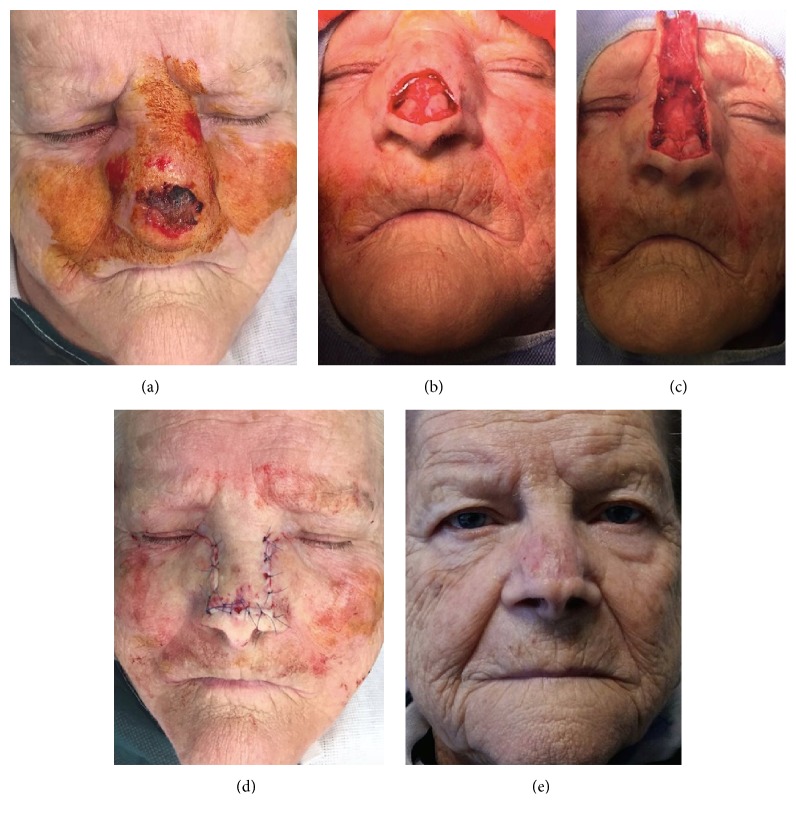
72-year-old patient affected by basal cell carcinoma involving the distal third area of the nose (a). Intraoperative image of the defect after tumor excision (b) and flap harvesting (c). Immediate postoperative (d) and postoperative result after 18-month follow-up. Picture showing no distortion of the profile of the nose and good texture.

**Figure 2:**
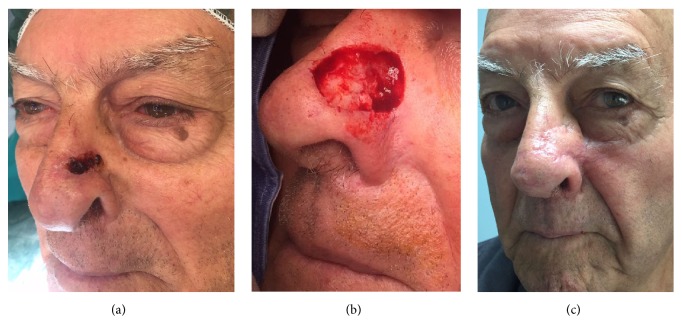
84-year-old patient affected by squamous cell carcinoma involving the medial third area of the nose and left sidewall (a). Intraoperative image of the defect after tumor excision (b). Patient underwent reconstruction with locoregional nasolabial flap. Postoperative picture after 12-month follow-up (c).

**Figure 3:**
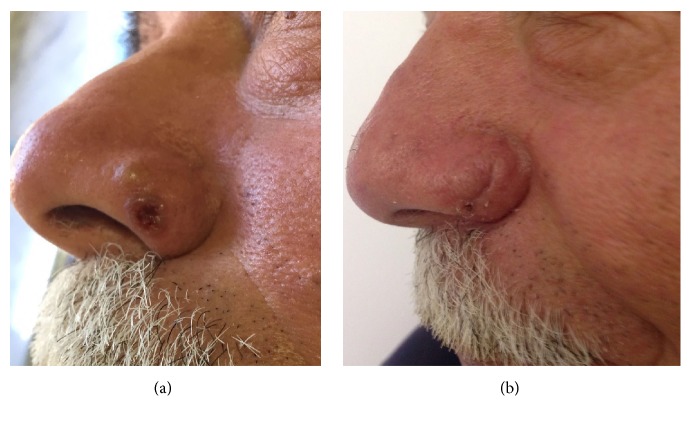
58-year-old patient affected by squamous cell carcinoma involving the left ala nasi (a). The lesion is very close to the inferior margin. Patient underwent reconstruction with locoregional rotational flap. Postoperative picture after 1-month follow-up shows no distortion of the ala. (b).

**Figure 4:**
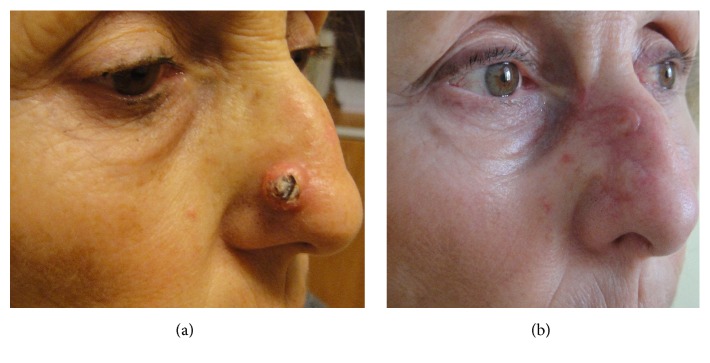
69-year-old patient affected by basal cell carcinoma involving the distal third area of the nose (a). Patient underwent reconstruction with locoregional bilobed flap. Postoperative result after 45 days. (b).

**Figure 5:**
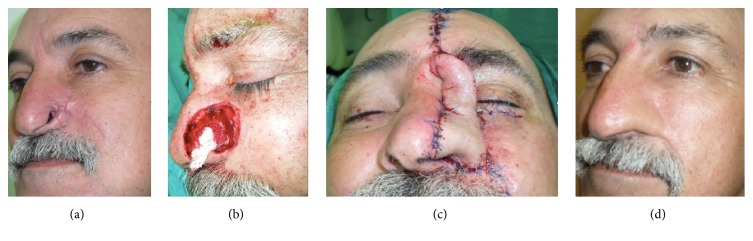
70-year-old patient affected by recurrent and infiltrating basal cell carcinoma previously treated by tumor excision and reconstruction with nasolabial flap. The resulting defect involves more than one subunit of the nose (a). Intraoperative image after tumor excision (b). Immediate postoperative result after reconstruction with forehead flap and direct closure of the donor site (c). Postoperative result after second surgical step at 18-month follow-up (d).

All procedures excluding those requiring a forehead flap were performed under local anesthesia and completed through reconstruction of the defects by local flaps, according to the principles of aesthetic nasal reconstruction (Figures 1–5) [8, 9].

All histology reports from nasal skin malignancies were evaluated and only NMSC cases with histopathological confirmation were included in this study. Nasal region was arranged into 3 groups according to the anatomical zones affected by the lesion: proximal, middle, and distal third. Then we included an additional fourth group for complex defects involving more than one subunit [10]. Postoperative results were documented by digital imaging.

## 3. Results

A total of 310 patients (203 males and 107 females) with diagnosis of NMSC (115 SCCs and 195 BSCs) were enrolled in the study after treatment by conventional tumor excision followed by surgical reconstruction by locoregional flap. Tumors excised through Mohs' surgery were not considered. After diagnosis confirmation, postoperative ultrasonography was performed in all SCC cases to assess lymphatic involvement and afterwards patients were referred to the oncology department. Follow-up was scheduled at 1, 3, 6, 12, 18, and 24 months. The distribution of nasal tumors on the different nasal subunits is outlined in Table 1.

71 defects were located in the proximal third, 56 in the middle third, 126 in the distal third, and 57 were identified as combined defects involving the proximal and middle thirds and the middle and distal third of the nose as well as the entire structure. In these cases, the initial tumor had already spread widely over more than one subunit. Reconstruction was performed with glabellar flap, bilobed flap, V-Y advancement flap, dorsal nasal or Miter flap, and nasolabial and forehead flap. The most frequent location was the nasal ala (24,1 percent), followed by the dorsum (20,9 percent), tip (16,4 percent), and sidewall (15,8 percent). Reconstruction with locoregional flaps was performed in all cases. 28 out of 36 patients who underwent forehead flap reconstruction received donor site direct suture. 2 of the remaining patients were skin grafted and the rest of them left to heal by secondary intention. Postoperative dressing was performed every 3–5 days, until complete wound healing or stitches removal that occurred between 7 and 21 postoperative days. Complete wound healing was achieved after an average of 11.8 days after first surgery.

7 patients (2,2 percent) developed complications requiring revision, such as partial flap necrosis (1 patient) and dehiscence of the wound or of the donor site closure (6 patients), occurring especially after forehead flaps reconstruction.

6 patients developed infection and 3 presented hematoma in the early postoperatory period, successfully managed by oral antibiotics administration or blood pressure control in association with partial suture removal and evacuation (2,9 percent). Cancer recurrence rate in our series was extremely low (5 recurrent cancers: 2 in the proximal third area, 1 in the middle third area, and 2 in the distal third area). In a review of 310 cases (1,6 percent), only 1 patient underwent reexcision (middle third area) and secondary flap reconstruction. For the rest of them, we advocated close surveillance and increased vigilance especially for squamous cancers.

## 4. Discussion

Most of the nasal defects presenting for reconstruction are secondary to tumor excision and the increasing incidence of nonmelanoma skin cancer goes along with our aging population and with a combination of different condition, such as sun exposure and ozone depletion [4, 11].

Nonmelanotic skin cancers (NMSCs) are the most common of all cancers, with over one million cases diagnosed annually in the US. NMSCs recurrence rates reported in the literature are from 9 to 30%, following surgical extirpation with clear perilesional margins, and among them SCCs are more liable to recur (54%), followed by BCCs (35%) [12, 13]. Even though much has been written in the literature regarding nasal reconstruction, the management of nasal defects can be often challenging and the best “reconstruction” is still to be found [4, 6, 14]. Plastic surgeons are often expected to reconstruct posttumoral defects immediately after their primary excision or Mohs' surgery, and sometimes that is the first time they see the patient [3, 4].

Major targets to be pursued when facing this kind of reconstructions should be maintenance of similar skin color and rebuilding of the nasal structural lining and support, in order to avoid airway stenosis.

Millard was the first to introduce the concept of aesthetic units in nasal reconstruction, describing how wound closure at the junctions between units would help to improve surgical results as well as cosmetic appearance of prior nasal reconstructions [3]. After this, Burget and Menick revolutionized nasal reconstruction concept advocating a strict anatomic approach and meticulous surgical technique while dividing the nose into multiple aesthetic subunits, in order to obtain a radical excision, improving the aesthetic result [8, 9]. Following this principle, if more than 50% of the subunit is apparently involved by the tumor, then surgical extirpation should extend to the whole subunit, resulting in a larger defect. Then reconstruction would be more challenging, requiring the sacrifice of a wider portion of healthy tissue [15]. Oncological clearance should be always pursued through a multidisciplinary approach, especially for large infiltrating tumors, although the extent of the residual defect after excision may result in technical difficulties for the reconstruction. For this reason, recently many authors have shown some concern about the Burget and Menick theory, especially in case of elderly patients, presenting multiple comorbidities and tumor margins difficult to visualize macroscopically, where a radical but conservative treatment could be preferred [3–6].

In this regard, locoregional flaps continue to play a substantial role in reconstruction of soft tissue and cutaneous defects of the head and neck [5]. The nasal pyramid is located on the middle face and because of its prominence and central location, is often related to behavior and personal identity [4]. Restoration of complex nasal defects that include loss of the lining, the subsurface framework, and the soft tissue coverage remains one of the most difficult problems presented to the reconstructive surgeon, due to the aesthetic importance of the nose. Considering this, advances in local and regional flap reconstruction have shown distinct advantages over the use of autologous skin graft or free tissue transfer in certain circumstances [5]. Several authors published different algorithms for nasal reconstruction after malignant tumor resection derived from analysis of their case series, with the aim of offering a simple, safe, and universal road-map for nasal reconstruction [2, 6, 10, 16, 17].

Patient comorbidities, location, size, shape, and orientation of the defects are important factors in determining the method used in reconstruction [6]. Age in association with other serious medical comorbidities can preclude multistage operations and smoker candidates to nasal reconstruction should quit smoking at least 2 weeks before surgery to preserve skin vascularity [3, 4]. In fairly superficial full-thickness as small partial nasal defects, full-thickness grafts may be acceptable, since they represent an easy, safe, and fast reconstructive solution, especially when temporary closure of the defect in expectation of definitive pathology is needed [2].

Nevertheless for defects larger than 1.5 to 2 cm in diameter, Rohrich et al. generally suggest the use of axial pattern flaps such as the forehead flap, the nasolabial flap, and the dorsal nasal flap [3, 6]. These methods of reconstruction can often be used interchangeably, but all of them show specific pearls and pitfalls. Certain flaps work better in different areas such as glabella, Miter for horizontal defects, and V-Y and nasolabial flaps for vertical loss of substance [6]. Dorsonasal flaps should be always developed in the deep submuscular plane above the periosteum in order to obtain sufficient laxity for the coverage of full-thickness defects of the nasal dorsum [6].

On the other hand when planning a reconstruction, it is useful to maintain a global approach to the defect and the surrounding tissues, keeping in mind that natural cheek laxity can provide a great advantage, especially in case of elderly patients with redundant tissue. In this regard, nasolabial flaps represent a good option offering excellent outcomes, either when harvested as superior or inferior based flap. Reviewing our experience we believe that including excessive fatty tissue should be avoided in order to prevent a “bumpy effect” and further thinning procedures. These flaps sometimes require two surgical stages for contour irregularities revision but, on the other hand, offer more reliable blood supply and tend not to distort the contour of the nose itself [3].

Among locoregional flaps, bilobed flap represents a valid reconstructive alternative. In this series, we report 31 bilobed flaps for coverage of defects of the distal third area of the nose with overall satisfactory results, representing a very useful, simple, and reliable technique. Nevertheless, in several cases, we registered a tendency to mild postoperative contour distortion of the nose, concavity at the donor site, or pin-cushioning at the recipient site, probably due to the violation of the subunit principle related to the technique itself.

When dealing with larger, multisubunit or even total nasal defects, forehead flaps represent the most used and performing option. It is traditionally described as two- or three-stage flap, in order to allow delayed flap defatting or revision. Alar defects as well as defects of the tip and dorsum larger than 2 cm are best treated with a tailored paramedian forehead flap. It is a relatively easy and safe flap, thanks to its reliable blood supply and skin color, texture, and pliability, which perfectly matches with the receiving area. When programming a paramedian forehead flap, a template is created before transferring the flap to cover the defect, usually the design is performed on the contralateral side aiming, when possible, to minimize involvement of the hair-bearing scalp. The forehead flaps are left in place for about 4 weeks postoperatively. In the meanwhile, donor sites, if not fully closed, can be skin grafted or partially or completely left to heal secondarily. Additional flap-thinning stage is common to reach satisfactory aesthetic outcomes. The characteristics of patient's defect and a deep understanding of vascular anatomy of the donor area should always guide surgeon during preoperative planning.

### 4.1. Proximal Third Area

In the proximal third zone small nasal defects were basically treated with glabellar flaps, Miter (dorsal nasal) flaps, and V-Y advancement flap. In case of combined defects, forehead flap was the first option. One dehiscence of a V-Y flap was experienced as well as 3 infections in case of glabellar and 1 hematoma in case of Miter flap performed in this area, requiring partial suture removal and evacuation.

The glabellar flap was considered the best choice when dealing with the reconstruction of superior or lateral area of the proximal subunit, while dorsal nasal flap was preferred in case of central proximal subunit lesions. These flaps show similar skin characteristics to the defect area, in terms of color, texture, and thickness.

### 4.2. Middle Third Area

In the middle third subunit, the defects can be covered either with a Miter, nasolabial, or V-Y advancement flap. We experienced 1 partial necrosis in case of a V-Y advancement flap and 1 dehiscence of the wound requiring revisions, as well as 2 cases of infection after performing middle third areas reconstructions.

In the central subunit, Miter flaps seem to be the best option alternative to direct linear closure. Depending on how laterally the defect is located, we can design a V-Y advancement flap onto the cheek, based on the perforators from the angular artery, or a nasolabial flap (Figure 2). In case of combined defects, forehead flaps remain the best option.

### 4.3. Distal Third Area

In the distal third zone, domal and alar defects can be covered with nasolabial flaps, V-Y advancement flaps, Miter flaps, bilobed flap, forehead flaps, or simple rotational flaps (Figures 3 and 4).

In case of combined defects of the distal third zone, forehead flaps represent the best option, followed by nasolabial and extended V-Y flaps (Figure 5).

In this area, we performed the highest number of forehead flap reconstructions.Three dehiscences of the donor site and forehead flap itself required revisions; 1 case of dehiscence, 1 case of infection, and 2 cases of hematoma occurred after performing reconstructions of the distal third area or combined defects of the nose.

V-Y flap should extend into the nasolabial region and are carefully designed right above the alar groove. The dorsal nasal flap can alternatively manage dorsal and tip defects from 1 to 2 cm. It usually can be considered in defects of the distal zone mainly horizontally oriented. It has the advantage of less local distortion than the bilobed flap. Limitations include defects that are close to the alar rim and those that extend over the domes [3, 18, 19]. Due to its versatility, bilobed flaps are preferred in case of central subunit and dome region, always paying attention to avoid distortion of the nasal tip, dorsal ridging, or depression and alar notching [20–22].

Rintala flap represents an alternative for distal third defects of the dorsal region (Figure 1). The columella defects are not very common but certainly the most challenging to reconstruct. Nasolabial flaps remain probably the best option. This is a one- or two-stage flap that can be designed as a random vascularized flap or even based on perforators [23].

## 5. Conclusion

Multiple surgical options exist for repairing of cutaneous defects involving the nose, and all of them must be part of the surgeon's armamentarium. Nevertheless, locoregional flaps remain a useful tool for nasal reconstruction after tumor excision and often provide unique characteristics not available with other surgical options. An accurate analysis of the defect combined with a very careful evaluation of the patients' clinical condition should be considered in order to select the best surgical option. Complete and exhaustive knowledge of locoregional vascular anatomy and a comprehensive multidisciplinary approach are essential preliminary steps for the correct treatment of nasal skin cancer. Radical tumor control and a satisfactory aesthetic and functional result are primary goals. Reconstructive surgeons should approach each patient as a distinct individual with a unique defect and perform the best reconstruction possible, tailored based on patient's needs and expectations. At the conclusion of the procedure and during the healing process, close clinical postoperative follow-up is mandatory, as well as the educating process of patients, to play an active role not only in the preoperatory decision-making but also during postoperative long-term self-care and skin control.

## Figures and Tables

**Table 1:** Patients characteristics, cancer histological type, and recurrence rate are reported. The table summarizes the localization of the lesions and relate it with the correspondent reconstructive option and locoregional flap. Postoperative surgical complications rate are pointed out.

Patients	Localization	Locoregional flap	Complications
(i) Number of patients: 310 (ii) Male: 203 (65.48%) (iii) Female: 107 (34,52%) (iv) Age: 72.28 ± 12.15 years (range 40–88 y)	Proximal third area *n* = 71 (22,9%)	(i) Miter (dorsonasal) Flap *n* = 20 (28,2%) (ii) Glabellar flap *n* = 32 (45,1%) (iii) Rotation flap *n* = 13 (18,3%) (iv) Others *n* = 6 (8,5%)	*Complications requiring revisions* Number of patients: 7 (2,3%) (i) Partial flap necrosis (*n* = 1) (ii) Dehiscence of the wound/donor site (*n* = 6)

*NMSC histological type* (i) Basal cell carcinoma (BCC): 195 (62,9%) (ii) Squamous cell carcinoma (SCC): 115 (37.1%)	Middle third area *n* = 56 (18,1%)	(i) Miter (dorsonasal) flap *n* = 15 (26,79%) (ii) Nasolabial flap *n* = 12 (21,43%) (iii) V-Y advancement flap *n* = 14 (25%) (iv) Rotation flap *n* = 10 (17,9%) (v) Others *n* = 5 (8,9%)	*General complications:* Number of patients: 9 (2,9%) (i) Infection (*n* = 6) (ii) Hematoma (*n* = 3)
*Cancer recurrence rate* (i) Number of patients: 5 (1,6%)	Distal third area *n* = 126 (40,6%)	(i) Miter (dorsonasal) flap *n* = 21 (16,7%) (ii) Nasolabial flap *n* = 34 (27%) (iii) V-Y advancement flap *n* = 18 (14,3%) (iv) Rotation flap *n* = 21 (16,6%) (v) Bilobed flap *n* = 31 (24,6%) (vi) Others *n* = 1 (0,8%)	
	Complex defects (involving more than 1 subunit) *n* = 57 (18,3%)	(i) Forehead flap *n* = 36 (63,1%) (ii) Nasolabial flap *n* = 12 (21,1%) (iii) V-Y flap *n* = 9 (15,7%)	
